# Learning engagement and psychological flexibility among Chinese adolescents: a moderated mediation model

**DOI:** 10.3389/fpsyg.2025.1407707

**Published:** 2025-03-06

**Authors:** Pengfei Yue, Jiaxin Zhang

**Affiliations:** ^1^College of Education Science, Hubei Normal University, Huangshi, China; ^2^Research Center for High-Quality Development of Basic Education, Huangshi, China; ^3^Faculty of Education and Arts, College of Arts and Science of Hubei Normal University, Huangshi, China

**Keywords:** learning engagement, harsh parenting, psychological flexibility, gender difference, acceptance and commitment therapy

## Abstract

**Background:**

Psychological flexibility is a key factor in maintaining mental wellbeing. While previous studies have used professional Acceptance and Commitment Therapy (ACT) interventions to enhance psychological flexibility, our study proposes a new approach to enhance psychological flexibility based on the Development-in-Sociocultural-Context Model for Childrenl Acceptance and Commitmen

**Methods:**

This study, adopting a positivist research approach, utilized a cross-sectional design. Data were collected from 750 students at three middle schools in Henan Province, China, through cluster random sampling. This approach yielded 750 questionnaires. The participants sequentially completed three questionnaires: the Utrecht Work Engagement Scale-Student (UWES-S), the Harsh Parenting Questionnaire, and the Avoidance and Fusion Questionnaire for Youth. After removing 45 invalid questionnaires due to incomplete responses and patterned answers, a total of 705 valid questionnaires were obtained, with 52.9% girls and an average age of 13.11 years (SD = 1.04), leading to an effectiveness rate of 94.00%. Upon data collection, SPSS 26.0 software was used for correlation analysis, mediation analysis, and moderated mediation analysis to assess the relationships between variables.

**Results:**

(1) Learning engagement positively predicts psychological flexibility; (2) harsh parenting mediates the relationship between learning engagement and psychological flexibility; and (3) gender moderates the impact of harsh parenting on psychological flexibility, with a more significant negative effect on females than males.

**Conclusion:**

This study highlights a novel approach for enhancing psychological flexibility among Chinese adolescents, demonstrating that by increasing their learning engagement, adolescents can effectively improve their psychological flexibility. Future research could investigate various cultural, age, and occupational groups to explore effective approaches for enhancing psychological flexibility in diverse populations, which is vital for promoting human mental wellbeing.

## Introduction

The Buddhist saying, “All beings are entangled in suffering,” poignantly captures a universal truth about the human condition. This acknowledgment of universal suffering provides a compelling backdrop for the study of psychological flexibility. Psychological flexibility is defined as the ability to be consciously in contact with the present moment, completely and without judging or trying to change the thoughts and emotions that occur at that moment, and persistently keeping in mind personal values and goals (Hayes et al., 2012). Research has demonstrated that psychological flexibility is instrumental in alleviating human suffering, playing a significant role in mitigating mental health issues. In the context of rapidly changing educational environments, psychological flexibility has emerged as a critical skill for adolescents to navigate academic pressures, social challenges, and emotional distress. Research indicates that higher levels of psychological flexibility are associated with better academic performance, reduced anxiety, and improved social relationships (Davis et al., 2020; Fonseca et al., 2020; Yue et al., 2021). Therefore, fostering psychological flexibility in adolescents is not only essential for their mental wellbeing but also for their long-term success in an increasingly complex world. This includes reducing the impact of anxiety (Arch et al., 2012; Davis et al., 2020), depression (Fonseca et al., 2020; Lappalainen et al., 2015), and substance addiction (Lanza et al., 2014). Thus, exploring methods to enhance psychological flexibility is of paramount importance.

Meta-analytic evidence suggests that the predominant approach to enhancing psychological flexibility has been through direct intervention using Acceptance and Commitment Therapy (ACT) (Ren et al., 2019). While effective, this method is not without its drawbacks. On one hand, it requires the expertise of professionals trained in ACT, which limits accessibility due to the insufficient number of trained ACT counselors to meet the demand. On the other hand, seeking help from ACT professionals can be a significant financial burden for many. This raises a critical question: Is there a more accessible and cost-effective approach to help individuals improve their psychological flexibility?

The Development-in-Sociocultural-Context Model for Children’s Engagement in Learning offers a guiding framework to explore this issue. This model posits that learning engagement can lead to a range of long-term benefits, such as improved psychosocial adjustment and reduced behavioral problems (Wang et al., 2019). This view is corroborated by extensive research. For example, studies show that at-risk or marginalized children who are deeply engaged in learning demonstrate greater adaptability in challenging situations (Reschly and Christenson, 2012). Conversely, a lack of engagement is linked to increased drug use and criminal behavior (Henry et al., 2012; Li and Lerner, 2011). Furthermore, ACT suggests that the root of many human psychosocial maladjustments, including depression and issues like substance abuse, lies in a deficit of psychological flexibility (Coto-Lesmes et al., 2020; Strosahl, 2004). Considering that both learning engagement and psychological flexibility are instrumental in addressing psychosocial and behavioral problems, a potential connection between the two becomes apparent.

**FIGURE 1:**
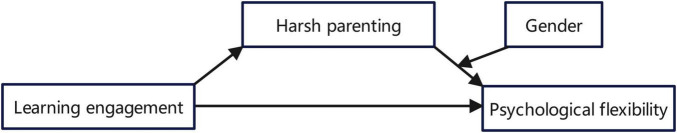
Proposed moderated mediation model.

Given this potential connection, our study builds upon the Development-in-Sociocultural-Context Model for Children’s Engagement in Learning and relevant empirical research to propose a moderated mediation model (Figure 1 for the proposed model). Recent studies have highlighted the role of psychological flexibility in adolescent mental health (Bibi et al., 2022; Yue et al., 2021). However, most research has focused on professional interventions such as ACT (Ren et al., 2019), which may not be accessible to all populations due to cost and resource limitations. This study proposes a novel approach by examining how learning engagement, a more accessible and cost-effective factor, can enhance psychological flexibility among adolescents. This model will enable us to explore the role of learning engagement in influencing psychological flexibility and to elucidate the underlying mechanisms of this relationship. By doing so, we aim to offer a novel and practical pathway for enhancing psychological flexibility.

## Learning engagement and psychological flexibility

Learning engagement refers to a sustained, positive state exhibited by students during the learning process, which is mainly characterized by vigor, dedication, and absorption (Schaufeli et al., 2002; Yue et al., 2022). Engagement in learning can be viewed as a training method to improve psychological flexibility. In the triadic model of ACT, Hayes et al. (2012) describe psychological flexibility in terms of three response styles: focus on the present moment (aware), openness to experience with acceptance and perspective on the events experienced (open), and commitment to value-directed action (active). When children are engaged in learning, their attention is fully focused on the present learning process (aware) rather than entangled in the content of private events (open). In addition, ACT asserts that values represent overarching qualities that individuals seek to express through ongoing actions (Hayes et al., 2012). In this context, engagement in learning is inherently a value that serves as a positive quality that children seek to manifest during the learning process and that guides their ongoing learning behaviors. Therefore, engaging in learning with vigor, dedication, and absorption is a value-directed action (active), and as a result, engaging in learning serves as an excellent practice for children to develop psychological flexibility. Previous empirical studies have found that there is a positive correlation between learning engagement and psychological flexibility (Martinie and Shankland, 2024).

Accordingly, this study proposes Hypothesis 1: Learning engagement positively predicts psychological flexibility.

## The mediating role of harsh parenting

According to the Development-in-Sociocultural-Context Model for Children’s Engagement in Learning, learning engagement not only directly bolsters an individual’s psychosocial adjustment but also indirectly enhances psychosocial adjustment through the augmentation of social support, leading to more favorable treatment from those around them (Wang et al., 2019). Given the Chinese cultural context, it is intuitive to consider the concept of “harsh parenting.” The traditional Chinese parenting belief, encapsulated in the saying “spare the rod, spoil the child,” often results in parents adhering to this philosophy treating their children sternly upon mistakes. For children, exhibiting higher learning engagement often translates to earning their parents’ commendation and experiencing gentler treatment, such as evading severe punishment for errors. This, in turn, indirectly boosts their psychological flexibility. Additionally, Bandura’s Triadic Reciprocal Determinism suggests that environmental factors (e.g., harsh parenting), individual factors (e.g., learning engagement), and behavioral outcomes (e.g., psychological flexibility) interact dynamically to shape adolescent development (Bandura, 1986). These theoretical frameworks provide a robust foundation for understanding the mechanisms through which learning engagement and harsh parenting influence psychological flexibility. In essence, this study seeks to elucidate the mediating role of harsh parenting in the nexus between learning engagement and psychological flexibility.

Firstly, learning engagement might inversely predict harsh parenting. Harsh parenting encompasses parents’ coarse behaviors, emotions, and attitudes toward their offspring (Wang et al., 2016). This includes not just overt verbal and physical aggression like yelling and hitting but also psychological affronts such as neglecting and dismissing children’s emotional needs, as well as coercive actions like infringing on children’s rights and meting out unwarranted punishments (Wang et al., 2016). The Transactional Model of Development posits a bidirectional relationship between a child’s temperament or behavioral display and parental rearing (Sameroff, 2009). As per this model, while children’s behavioral manifestations are influenced by parental rearing, they also reciprocally impact it. Prior research indicates that children exhibiting pronounced externalized behavioral issues or heightened anger temperaments are more prone to elicit corporal or authoritarian parenting from their guardians (Lee et al., 2013). Conversely, commendable behavior in children can mitigate tendencies toward parental harshness (Sameroff, 2009). Given the emphasis Chinese parents traditionally place on academic achievement, children’s pronounced learning engagement is likely to curtail harsh parenting.

Secondly, harsh parenting might inversely predict psychological flexibility. Past studies have underscored that the parenting style is a pivotal determinant of a child’s psychological flexibility (Flujas-Contreras and Gómez, 2018; Williams et al., 2012). For instance, an individual raised by critical parents may become excessively self-critical and shun intimacy fearing reproach in a bid to conform to parental expectations, and, persistently entangled with these negative cognitions while evading social interactions, would exhibit compromised psychological flexibility (Hayes et al., 2012). It is plausible that children frequently subjected to harsh parenting often act based on their parents’ preferences or values, rather than making autonomous, situationally adaptable decisions. In such a stringent parenting milieu, children’s psychological flexibility is gravely jeopardized. In empirical research, both Bibi et al. (2022) and Yue et al. (2021) found that parental control and harsh parenting negatively impact adolescents’ psychological flexibility.

Consequently, this study proposes Hypothesis 2: Harsh parenting mediates the relationship between learning engagement and psychological flexibility.

## The moderating role of gender

The influence of harsh parenting on psychological flexibility may exhibit gender disparities. Studies have indicated that individuals frequently subjected to severe parenting methods, such as physical punishment, verbal reprimands, and punitive measures, tend to manifest negative emotions, including anxiety and depression (Pineda et al., 2007; Yue et al., 2022). Compared to males, females are more predisposed to employ experiential avoidance as a coping mechanism to mitigate the negative emotions stemming from stressful events, such as harsh parenting (Panayiotou et al., 2017; Venta et al., 2012). Notably, experiential avoidance is a significant precursor to psychological inflexibility (Hayes et al., 2012). Consequently, harsh parenting might exert a more pronounced detrimental effect on the psychological flexibility of females.

In light of these observations, this study proposes Hypothesis 3: Gender moderates the impact of harsh parenting on psychological flexibility, with a more significant negative effect on females than males.

## Materials and methods

### Research design

The research approach of this study is based on positivism. A cross-sectional design was used to conduct a questionnaire survey on participants. They sequentially completed three questionnaires: the Utrecht Work Engagement Scale-Student (UWES-S), the Harsh Parenting Questionnaire, and the Avoidance and Fusion Questionnaire for Youth. After data collection, correlation analysis, mediation analysis, and moderated mediation analysis were employed to examine the relationships between variables.

### Participants

In this study, data were collected at a single time point from 750 students at three middle schools in Henan Province, China, using cluster random sampling, resulting in 750 questionnaires for the survey. After excluding 45 invalid questionnaires due to incomplete responses and patterned answering, 705 valid questionnaires were obtained, resulting in an effectiveness rate of 94.00%. Among the valid responses, the average age of participants was 13.11 years (SD = 1.04). The sample comprised 332 boys (47.1%) and 373 girls (52.9%). In terms of grade distribution, 349 students (49.5%) were in the 7th grade, 177 (25.1%) in the 8th grade, and 179 (25.4%) in the 9th grade. The sample included 44 only children (6.2%) and 661 students with siblings (93.8%). Furthermore, 597 participants (84.7%) hailed from rural backgrounds, while 108 students (15.3%) were from urban areas. To provide a better picture of the demographic profile, we have presented the details in Table 1.

**TABLE 1:** Participant demographics.

Variable	Category	Frequency	Percentage
Gender	Male	332	47.1%
	Female	373	52.9%
Grade	7th grade	349	49.5%
	8th grade	177	25.1%
	9th grade	179	25.4%
Only child status	Only child	44	6.2%
	Non-only child	661	93.8%
Family location	Rural	597	84.7%
	Urban	108	15.3%

### Research tools

#### Utrecht Work Engagement Scale-Student

The UWES-S compiled by Schaufeli et al. (2002) and revised by Fang et al. (2008) was adopted. The questionnaire has been proved to have good reliability and validity in the Chinese context. It has 17 items (such as “As far as my studies are concerned, I always persevere, even when things do not go well”) and three dimensions, namely vigor, dedication and absorption. Using the 7-point scoring method, the higher the score, the higher the level of individual learning engagement. In previous studies on learning engagement, the scale showed good psychometric characteristics (Li, 2018; Zhang and Yue, 2021). In this study, the Cronbach’s α of this scale was 0.96.

#### Harsh parenting questionnaire

Referring to the research of Wang (2017) and Qi et al. (2020), four subjects were used to measure parental harsh parenting. The questionnaire has been proved to have good reliability and validity in the Chinese context. Topics such as “When I did something wrong or made my parents angry, he (or she) would lose temper or even yell at me,” asking teenagers to evaluate their parents. Using the 5-point scoring method, the higher the score, the higher the degree of harsh parenting. In this study, Cronbach’s α of the harsh parenting questionnaire was 0.79.

#### Avoidance and fusion questionnaire for youth

The Avoidance and Fusion Questionnaire for Youth (simplified version) (AFQ-Y8) prepared by Greco et al. (2008) and revised by Chen et al. (2019) was adopted. The questionnaire has been proved to have good reliability and validity in the Chinese context. It questionnaire has 8 items (such as “My life won’t be good until I feel happy”), one dimension. The 5-point scoring method is adopted. 1 means “completely non-conforming” and 5 means “fully conforming;” Reverse scoring indicates the degree of individual psychological flexibility, the higher the score, the higher the degree of psychological flexibility. In previous studies on adolescents’ psychological flexibility, the questionnaire showed good psychometric characteristics (Liu et al., 2021). The Cronbach’s α of this scale in the present study was 0.81.

#### Procedure

Before the questionnaire phase, moral education instructors at participating schools conducted thorough training for class head teachers about the administration of the test and key precautions. The head teachers then briefed parents about the test, leading to the voluntary signing of informed consent forms by both parents and students. During the test, students filled out paper questionnaires under the guidance of head teachers during class meetings, focusing on honest and reflective responses in line with their real experiences. Emphasis was placed on the confidentiality of their responses. The questionnaire completion took around 20 min, after which the head teachers collected and securely maintained the questionnaires. The study adhered to the principles of the Declaration of Helsinki and was approved by the Research Ethics Committee at the College of Education Science, Hubei Normal University.

### Data analysis

The collected data were inputted and managed using SPSS 26.0 software, with which descriptive statistical analysis and correlation analysis were conducted. After standardizing the scores of each scale, the two models under investigation were tested using the PROCESS macro program. Specifically, Model 4 examined the mediation effect of harsh parenting, while Model 14 was applied to explore the moderating effect of gender.

## Results

### Common method biases test

To mitigate the risk of common method biases inherent in self-reported data, strategies such as the use of anonymous surveys and the inclusion of reverse-scored items were adopted. Moreover, the validity of the data was further assessed through the application of the Harman single factor test. The result of unrotated exploratory factor analysis were shown that the first factor only can explain 38.60% variance, which was less than the 40% critical standard (Podsakoff et al., 2003), indicating that there were no serious common method biases in this study.

### Preliminary analyses

The mean and standard deviation for each of the variables and the correlation coefficients between them are shown in Table 2. Table 2 shows that learning engagement was significantly negatively correlated with harsh parenting and positively correlated with psychological flexibility; there was a significant negative correlation between harsh parenting and psychological flexibility. In addition, grade, whether the only child or not were significantly correlated with harsh parenting, family location was significantly correlated with learning engagement, and the correlation between gender, age and research variables was not significant. Therefore, in the follow-up analysis, grade, whether the only child and family location were treated as control variables.

**TABLE 2:** Descriptive statistics and correlation analysis results for each variable (*n* = 705).

Variables	M ± SD	1	2	3	4
1 Learning engagement	3.78 ± 1.40	1			
2 Harsh parenting	1.77 ± 0.79	−0.21**	1		
3 Psychological flexibility	3.35 ± 0.85	0.22**	−0.28**	1	

**p < 0.01.

### Learning engagement and psychological flexibility: moderated mediation effect

According to the suggestions of Hayes (2013) and Wen and Ye (2014), all variables were standardized, and then an analysis of moderated mediation effect was carried out under the control of grade, only child or not and family location. All analysis processes were carried out by SPSS 26.0 macro program PROCESS. The percentile bootstrap method with deviation correction was used for inspection. Repeated sampling for 5,000 times to calculate the 95% confidence interval (see Table 3 for the specific results).

**TABLE 3:** Learning engagement and psychological flexibility: moderated mediation effect.

Predictive variable	Harsh parenting			Psychological flexibility			Psychological flexibility		
	**β**	**SE**	**t**	**β**	**SE**	**t**	**β**	**SE**	**t**
Learning engagement	−0.20	0.04	−5.32**	0.17	0.04	4.57**			
Harsh parenting				−0.24	0.04	−6.63**	−0.31	0.05	−6.38**
Gender							0.09	0.07	1.21
Harsh parenting × gender							0.15	0.07	2.07*
*R* ^2^		0.07			0.11			0.11	
*F*		12.23**			16.45**			12.65**	

**p < 0.01.

The first step was to test the simple mediation model (model 4). Regression analysis showed that learning engagement had a significant positive predictive effect on psychological flexibility (β = 0.22, *p*< 0.01); After incorporating harsh parenting into the regression equation, learning engagement still had a significant positive predictive effect on psychological flexibility (β = 0.17, *p* < 0.01), learning engagement negatively predicted harsh parenting (β = −0.20, *p* < 0.01), harsh parenting negatively predicted psychological flexibility (β = −0.24, *p* < 0.01). a × b = 0.05, boot SE = 0.01, 95% confidence interval was (0.03, 0.07), excluding 0. This result showed that harsh parenting played a significant mediating role between learning engagement and psychological flexibility, and the mediating effect accounts for 22.22% of the total effect.

The second step was to test the moderated mediation effect (model 14). Regression analysis showed that harsh parenting negatively predicted psychological flexibility (β = −0.31, *p* < 0.01), gender had no significant predictive effect on psychological flexibility (β = 0.09, *p* = 0.23), and the interaction between harsh parenting and gender had a significant predictive effect on psychological flexibility (β = 0.15, *p*< 0.05), 95% confidence interval was (0.01, 0.29), excluding 0. This result showed that gender played a moderating role in the relationship between harsh parenting and psychological flexibility.

**FIGURE 2:**
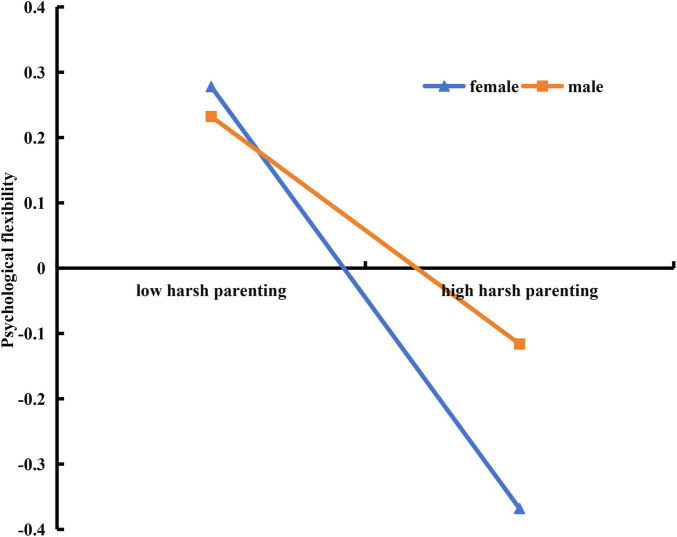
Gender as a moderator in the relationship between harsh parenting and psychological flexibility.

In order to reveal how gender moderated the impact of harsh parenting on psychological flexibility, gender was divided into males and females, a simple slope test was performed, and a simple effect analysis chart was drawn (Figure 2). The results showed that harsh parenting had a stronger impact on females’ psychological flexibility (b simple = −0.34, *p* < 0.01), and the 95% confidence interval was (−0.43, −0.24); It had a weaker effect on male’s psychological flexibility (b simple = −0.19, *p* < 0.01), and the 95% confidence interval was (−0.30, −0.08).

## Discussion

### Learning engagement and psychological flexibility

The finding that learning engagement positively predicts psychological flexibility aligns with previous research suggesting that active participation in learning activities fosters adaptive psychological responses (Lawson and Lawson, 2013). However, the mediating role of harsh parenting highlights the importance of family dynamics in shaping adolescent psychological flexibility, a finding that extends the current literature on parenting styles (Bibi et al., 2022; Yue et al., 2021). These results have practical implications for educators and parents, suggesting that interventions aimed at enhancing learning engagement and reducing harsh parenting practices could significantly improve adolescents’ psychological flexibility and overall mental wellbeing. This study is pioneering in its discovery that learning engagement positively predicts psychological flexibility. This finding aligns with the perspectives of the Development-in-Sociocultural-Context Model for Children’s Engagement in Learning and supports Hypothesis 1 (Wang et al., 2019).

By integrating the model with relevant empirical research, our study validates that learning engagement not only directly influences psychosocial adjustment and problematic behaviors but also directly impacts psychological flexibility. This discovery enriches the width of this model. While previous studies have used professional ACT interventions to enhance psychological flexibility (Ren et al., 2019), similar to the idea that meditation is not limited to temples, our study proposes that a novel approach to enhancing psychological flexibility is to increase children’s engagement in learning. It underscores that children can actively harness their agency, striving to increase their learning engagement, which in turn bolsters their psychological flexibility and overall mental wellbeing.

### The mediating role of harsh parenting

A significant contribution of this study is the identification of harsh parenting as a mediator between learning engagement and psychological flexibility. This finding aligns with the Perspectives of the Development-in-Sociocultural-Context Model for Children’s Engagement in Learning and supports Hypothesis 2 (Wang et al., 2019).

In the initial phase of the mediation process, our study revealed a negative association between learning engagement and harsh parenting. This suggests that individuals can mitigate their parents’ harsh behaviors through enhanced learning engagement, corroborating the predictions of the Transactional Model of Development (Sameroff, 2009). Prior research has indicated that children exhibiting pronounced externalized behavioral issues or heightened anger temperaments are more prone to elicit corporal or authoritarian parenting from their guardians (Lee et al., 2013). Conversely, commendable behavior in children can counteract tendencies toward parental harshness. This underscores that parents’ treatment of their children is indeed influenced by the children’s behavioral manifestations, with parents inclined to encourage positive behaviors and penalize negative ones. Moreover, studies focusing on school environments have found that students with higher learning engagement levels are more likely to garner respect from teachers and are more apt to receive support (Pitzer and Skinner, 2017). These evidences suggest that individuals with higher levels of learning engagement are more likely to receive better treatment in various contexts.

In the subsequent phase of the mediation process, our study found that harsh parenting negatively predicted psychological flexibility, resonating with prior research findings (Bibi et al., 2022; Yue et al., 2021). Adverse parenting styles can undermine an individual’s psychological flexibility. When parents fail to provide children with ample space for exploration, limiting their autonomy, children often struggle to make independent decisions based on situational demands, leading to diminished psychological flexibility.

It is worth noting that some readers might express skepticism regarding the proposed model’s validity, as it seemingly diverges from mainstream perspectives (e.g., the influence of harsh parenting on learning engagement). However, this deviation is precisely what sets this study apart. Triadic Reciprocal Determinism posits an interactive, Mutually causal relationship between environmental factors, individual factors, and behavior (Bandura, 1986). Based on this theory, we believe that the pathway where behavior (learning engagement) impacts individual factors (psychological flexibility) through the mediation of environmental factors (harsh parenting) is plausible. Moreover, our model, grounded in the perspectives of the Development-in-Sociocultural-Context Model for Children’s Engagement in Learning (Wang et al., 2019), is buttressed by empirical research at every juncture, underscoring the reliability of our assertions.

### The moderating role of gender

Our study identified gender as a significant moderator in the relationship between harsh parenting and psychological flexibility. Specifically, harsh parenting emerged as a more potent negative predictor of psychological flexibility for females compared to males, thereby validating Hypothesis 3.

This finding is in line with prior research, which has shown that negative parenting styles, such as authoritarian parenting, tend to have a more adverse impact on females’ psychological flexibility (Bibi et al., 2022). One potential explanation for this gender disparity lies in cultural norms prevalent in Asian societies. Historically, girls in these cultures have been granted less autonomy in decision-making compared to boys. Under the stringent control of authoritarian parents, girls often face restrictions that limit their ability to make flexible behavioral choices, compelling them to conform to parental directives (Bakhla et al., 2013). Consequently, their psychological flexibility becomes more susceptible to the negative influences of such parenting styles. Harsh parenting, characterized by controlling behaviors, often manifests in ways such as undue interference in children’s affairs, a lack of reasoned guidance, exaggerated reactions, or opting for forceful measures to compel compliance (Callahan et al., 2011; Rhoades et al., 2011). Given these dynamics, it is understandable why girls, who are already conditioned to be more compliant and less autonomous, would experience a more pronounced negative impact on their psychological flexibility due to harsh parenting.

### Limitations and future recommendations

Although this study is grounded in solid theory and prior research, it has certain limitations. Firstly, the use of a cross-sectional design precludes making definitive causal inferences about the relationships between variables. Secondly, our research adopts a variable-centered perspective, which does not account for potential heterogeneity within the subject population. Thirdly, as the participants were exclusively Chinese adolescents, the applicability of our findings to other populations requires further verification.

Future research could address these limitations in several ways. First, longitudinal designs or experimental methods could be employed to better examine the causal relationships between variables. Second, adopting a person-centered perspective could allow for a fuller consideration of heterogeneity within the study population, exploring the characteristics and influencing factors of psychological flexibility in different subtypes of subjects. Finally, future research could investigate various cultural, age, and occupational groups to explore effective approaches for enhancing psychological flexibility in diverse populations.

### Research implications

This study makes several contributions to the field of psychology, particularly in understanding how learning engagement can enhance psychological flexibility among adolescents. Firstly, it expands the Development-in-Sociocultural-Context Model for Children’s Engagement in Learning by integrating it with empirical findings on psychological flexibility. This integration provides a more comprehensive view of how learning behaviors and environments influence psychological flexibility. Secondly, the study sheds light on the mediating role of harsh parenting in the relationship between learning engagement and psychological flexibility, offering insights into family impact on adolescent development. Furthermore, the identification of gender as a significant moderating variable in this relationship deepens our comprehension of individual differences in psychological development. These insights have significant implications for targeted educational and family interventions to boost psychological health in Chinese adolescents, providing a foundation for further research in diverse contexts.

## Conclusion

Our study constructs a moderated mediation model to explore how learning engagement influences psychological flexibility in Chinese adolescents, revealing the significant roles of harsh parenting and gender differences. This research offers insights into the effects of learning behaviors and family interactions on adolescent development, underscoring the need for specialized educational and familial interventions to improve mental wellbeing. These findings provide valuable guidance for future research to develop strategies that foster psychological flexibility across varied demographic groups.

## Data Availability

The raw data supporting the conclusions of this article will be made available by the authors, without undue reservation.

## References

[B1] Arch J. J. Eifert G. H. Davies C. Vilardaga J. C. P. Rose R. D. Craske M. G. (2012). Randomized clinical trial of cognitive behavioral therapy (CBT) versus acceptance and commitment therapy (ACT) for mixed anxiety disorders. *J. Consult. Clin. Psychol.* 80 750–765. 10.1037/a0028310 22563639 PMC4718567

[B2] Bakhla A. K. Sinha P. Sharan R. Binay Y. Verma V. Chaudhury S. (2013). Anxiety in school students: Role of parenting and gender. *Ind. Psychiatry J.* 22 131–137. 25013314 10.4103/0972-6748.132927 PMC4085805

[B3] Bandura A. (1986). *Social Foundations of Thought and Action: A Social Cognitive Theory.* Hoboken, NJ: Prentice Hall.

[B4] Bibi A. Hayat R. Hayat N. Zulfiqar S. Shafique N. Khalid M. A. (2022). Impact of parenting styles on psychological flexibility among adolescents of Pakistan: A cross-sectional study. *Child Adolesc. Soc. Work J.* 39 313–322.

[B5] Callahan K. L. Scaramella L. V. Laird R. D. Sohr-Preston S. L. (2011). Neighborhood disadvantage as a moderator of the association between harsh parenting and toddler-aged children’s internalizing and externalizing problems. *J. Fam. Psychol.* 25 68–76. 10.1037/a0022448 21355648 PMC3071255

[B6] Chen Y. H. Zhao Y. Duan Y. M. Bai X. Y. Wang S. J. Zhu Z. H. (2019). Validity and reliability of the Chinese version of the avoidance and fusion questionnaire for youth (AFQ-Y8). *Chin. J. Clin. Psychol.* 27 1192–1195.

[B7] Coto-Lesmes R. Fernández-Rodríguez C. González-Fernández S. (2020). Acceptance and commitment therapy in group format for anxiety and depression: A systematic review. *J. Affect. Disord.* 263 107–120. 31818766 10.1016/j.jad.2019.11.154

[B8] Davis A. K. Barrett F. S. Griffiths R. R. (2020). Psychological flexibility mediates the relations between acute psychedelic effects and subjective decreases in depression and anxiety. *J. Contextual Behav. Sci.* 15 39–45. 10.1016/j.jcbs.2019.11.004 32864325 PMC7451132

[B9] Fang L. T. Shi K. Zhang F. H. (2008). Research on reliability and validity of utrecht work engagement scale-student. *Chin. J. Clin. Psychol.* 16 618–620. 10.3389/fpsyg.2024.1459362 39351103 PMC11439773

[B10] Flujas-Contreras J. M. Gómez I. (2018). Improving flexible parenting with acceptance and commitment therapy: A case study. *J. Contextual Behav. Sci.* 8 29–35. 10.1093/jpepsy/jsq118 21325269

[B11] Fonseca S. Trindade I. A. Mendes A. L. Ferreira C. (2020). The buffer role of psychological flexibility against the impact of major life events on depression symptoms. *Clin. Psychol.* 24 82–90.

[B12] Greco L. A. Lambert W. Baer R. A. (2008). Psychological inflexibility in childhood and adolescence: Development and evaluation of the avoidance and fusion questionnaire for youth. *Psychol. Assess.* 20 93–102. 10.1037/1040-3590.20.2.93 18557686

[B13] Hayes A. F. (2013). Introduction to mediation, moderation, and conditional process analysis. *J. Educ. Meas.* 51 335–337.

[B14] Hayes S. C. Strosahl K. D. Wilson K. G. (2012). *Acceptance and Commitment Therapy: The Process and Practice of Mindful Change.* New York: Guilford Press.

[B15] Henry K. L. Knight K. E. Thornberry T. P. (2012). School disengagement as a predictor of dropout, delinquency, and problem substance use during adolescence and early adulthood. *J. Youth Adolesc.* 41 156–166. 10.1007/s10964-011-9665-3 21523389 PMC4516271

[B16] Lanza P. V. García P. F. Lamelas F. R. González-Menéndez A. (2014). Acceptance and commitment therapy versus cognitive behavioral therapy in the treatment of substance use disorder with incarcerated women. *J. Clin. Psychol.* 70 644–657. 10.1002/jclp.22060 24449031

[B17] Lappalainen P. Langrial S. Oinas-Kukkonen H. Tolvanen A. Lappalainen R. (2015). Web-based acceptance and commitment therapy for depressive symptoms with minimal support: A randomized controlled trial. *Behav. Modification* 39 805–834. 10.1177/0145445515598142 26253644

[B18] Lawson M. A. Lawson H. A. (2013). New conceptual frameworks for student engagement research, policy, and practice. *Rev. Educ. Res.* 83 432–479.

[B19] Lee E. H. Zhou Q. Eisenberg N. Wang Y. (2013). Bidirectional relations between temperament and parenting styles in Chinese children. *Int. J. Behav. Dev.* 37 57–67. 10.1177/0165025412460795 23482684 PMC3591485

[B20] Li Y. Z. (2018). The impact of parental rearing style on learning engagement among senior high school students: A serial mediation effect model. *Psychol. Dev. Educ.* 34 576–585.

[B21] Li Y. Lerner R. M. (2011). Trajectories of school engagement during adolescence: Implications for grades, depression, delinquency, and substance use. *Dev. Psychol.* 47 233–247. 10.1037/a0021307 21244162

[B22] Liu Z. D. Bai X. Y. Zhang Y. Wu M. X. Liu Y. H. Zhu Z. H. (2021). Psychological flexibility training for career adaptability improvement among second-year middle-school students. *Chin. J. School Health* 42 399–403.

[B23] Martinie M. A. Shankland R. (2024). Achievement goals, self-efficacy, and psychological flexibility as antecedents of study engagement. *Soc. Psychol. Educ.* 27 2395–2416.

[B24] Panayiotou G. Karekla M. Leonidou C. (2017). Coping through avoidance may explain gender disparities in anxiety. *J. Contextual Behav. Sci.* 6 215–220. 10.1080/00224499.2024.2394827 39207062

[B25] Pineda A. Q. Cole D. A. Bruce A. E. (2007). Mother-adolescent interactions and adolescent depressive symptoms: A sequential analysis. *J. Soc. Pers. Relationsh.* 24 5–19.

[B26] Pitzer J. Skinner E. (2017). Predictors of changes in students’ motivational resilience over the school year: The roles of teacher support, self-appraisals, and emotional reactivity. *Int. J. Behav. Dev.* 41 15–29.

[B27] Podsakoff P. M. MacKenzie S. B. Lee J. Y. Podsakoff N. P. (2003). Common method biases in behavioral research: A critical review of the literature and recommended remedies. *J. Appl. Psychol.* 88 879–903. 10.1037/0021-9010.88.5.879 14516251

[B28] Qi D. Lin Y. Liu Q. X. (2020). Smartphone addiction out of “beating”? The effect of harsh parenting on smartphone addiction of adolescents. *Psychol. Dev. Educ.* 36 677–685.

[B29] Ren Z. H. Zhao C. X. Bian C. Zhu W. Z. Jiang G. R. Zhu Z. H. (2019). Mechanisms of the acceptance and commitment therapy: A meta-analytic structural equation model. *Acta Psychol. Sin.* 51 662–676.

[B30] Reschly A. L. Christenson S. L. (2012). “Jingle, jangle, and conceptual haziness: Evolution and future directions of the engagement construct,” in *Handbook of Research on Student Engagement*, eds Christenson S. L. Reschly A. L. Wylie C. (Berlin: Springer), 3–19.

[B31] Rhoades K. A. Leve L. D. Harold G. T. Neiderhiser J. M. Shaw D. S. Reiss D. (2011). Longitudinal pathways from marital hostility to child anger during toddlerhood: Genetic susceptibility and indirect effects via harsh parenting. *J. Fam. Psychol.* 25 282–291. 10.1037/a0022886 21480707 PMC3084154

[B32] Sameroff A. (2009). *The Transactional Model of Development: How Children and Contexts Shape Each Other.* Washington, DC: American Psychological Association.

[B33] Schaufeli W. B. Salanova M. González-Romá V. Bakker A. B. (2002). The measurement of engagement and burnout: A two sample confirmatory factor analytic approach. *J. Happ. Stud.* 3 71–92.

[B34] Strosahl K. D. (2004). “ACT with the multi-problem client,” in *A Practical Guide to Acceptance and Commitment Therapy*, eds Hayes S. C. Strosahl K. D. (Oakland, CA: New Harbinger), 209–244.

[B35] Venta A. Sharp C. Hart J. (2012). The relation between anxiety disorder and experiential avoidance in inpatient adolescents. *Psychol. Assess.* 24 240–248. 21895380 10.1037/a0025362

[B36] Wang M. T. Degol J. L. Henry D. A. (2019). An integrative development-in-sociocultural-context model for children’s engagement in learning. *Am. Psychol.* 74 1086–1102. 31829690 10.1037/amp0000522

[B37] Wang M. Z. (2017). Harsh parenting and peer acceptance in Chinese early adolescents: Three child aggression subtypes as mediators and child gender as moderator. *Child Abuse Neglect* 63 30–40. 10.1016/j.chiabu.2016.11.017 27902950

[B38] Wang M. Z. Du X. X. Zhou Z. K. (2016). Harsh parenting: Meaning, influential factors and mechanisms. *Adv. Psychol. Sci.* 24 379–391.

[B39] Wen Z. L. Ye B. J. (2014). Different methods for testing moderated mediation models: Competitors or backups? *Acta Psychol. Sin.* 46 714–726.

[B40] Williams K. E. Ciarrochi J. Heaven P. C. (2012). Inflexible parents, inflexible kids: A 6-year longitudinal study of parenting style and the development of psychological flexibility in adolescents. *J. Youth Adolesc.* 41 1053–1066. 10.1007/s10964-012-9744-0 22311519

[B41] Yue P. F. Hu W. L. Zhang M. (2021). The effect of harsh parenting on academic procrastination: The chain mediating effect of mental flexibility and state anxiety. *Chin. J. Special Educ.* 10 85–90.

[B42] Yue P. F. Hu W. L. Zhang J. X. Shi M. M. (2022). Harsh parenting and learning engagement among middle school students: The role of state anxiety and gender. *Stud. Psychol. Behav.* 20 226–232.

[B43] Zhang J. X. Yue P. F. (2021). The influence of harsh parenting on middle school students’ learning engagement: The mediating effect of perceived self-efficacy in managing negative affect and the moderating effect of mindfulness. *Psychology* 12 1473–1489.

